# Safety and efficacy of percutaneous radiofrequency thermocoagulation with varying puncture trajectories for recurrent maxillary trigeminal neuralgia: a prospective study

**DOI:** 10.3389/fneur.2025.1577399

**Published:** 2025-07-08

**Authors:** Yuanyuan Du, Xiaohui Liu, Hongchen Shi, Guangzhao Liu, Yue Ren, Lulu Xi

**Affiliations:** ^1^Department of Neurology, Second Hospital of Hebei Medical University, Shijiazhuang, China; ^2^Department of Pain, Second Hospital of Hebei Medical University, Shijiazhuang, China

**Keywords:** trigeminal neuralgia, maxillary nerve, foramen ovale, pterygopalatine fossa, recurrent, percutaneous radiofrequency thermocoagulation

## Abstract

**Background:**

Recurrence of maxillary trigeminal neuralgia (TN) poses a significant clinical challenge. Percutaneous radiofrequency thermocoagulation (PRT) has been established as an effective treatment for maxillary TN. However, the safety and efficacy of different puncture pathways, particularly in the context of pain recurrence, may vary.

**Objective:**

This study aimed to compare the safety and efficacy of PRT via the foramen ovale (FO) and pterygopalatine fossa (PF) approaches for recurrent maxillary TN.

**Methods:**

This prospective, non-randomized controlled study included 46 patients with recurrent maxillary TN who underwent PRT at our hospital between January 2021 and June 2021. Patients were divided into two groups based on the puncture pathway: the FO group and the PF group. All procedures were performed under local anesthesia and guided by computed tomography (CT). Operative complications were monitored, and pain was assessed using the Visual Analog Scale (VAS) and the Barrow Neurological Institute (BNI) scale. Follow-up evaluations were conducted at 6, 12, 18, and 24 months postoperatively.

**Results:**

The efficacy rates of the two puncture pathways within 24 months were 69.5% (FO group) and 78.2% (PF group), respectively. All patients experienced hypoesthesia in the maxillary nerve area. No severe complications, such as blindness, intracranial hemorrhage, or intracranial infection, were observed in either group.

**Conclusion:**

Both the FO and PF puncture pathways for PRT are safe and effective for treating recurrent maxillary TN. However, for patients with a history of prior PRT or percutaneous balloon compression (PBC) targeting the Gasserian ganglion, the PF approach may be preferable.

## Introduction

Trigeminal neuralgia (TN), characterized by excruciating paroxysmal facial pain, represents one of the most severe neuropathic pain conditions. Epidemiological studies report TN prevalence rates ranging from 4 to 29 cases per 100,000 person-years (1). Current therapeutic strategies encompass pharmacotherapy with antiepileptic drugs (AEDs) such as carbamazepine and oxcarbazepine, which demonstrate favorable initial efficacy but diminishing returns over time. For refractory cases, minimally invasive and surgical interventions—including percutaneous radiofrequency thermocoagulation (PRT), percutaneous balloon compression (PBC), microvascular decompression (MVD), and gamma knife radiosurgery (GKRS)—have shown substantial clinical benefits (2). While MVD remains the gold standard for addressing neurovascular conflict (NVC), its invasive nature and perioperative risks, particularly in elderly populations, limit universal applicability (3). Notably, longitudinal studies indicate eventual recurrence even after MVD with sufficient follow-up duration (4). Alternative modalities also exhibit significant relapse rates: PRT demonstrates 46% recurrence within 5 years, while PBC shows 19.2% recurrence (5, 6).

PRT has gained prominence as a viable treatment option for TN recurrence following failed primary therapies (7, 8). Anatomical targeting varies by pain distribution, with the foramen ovale (FO) and pterygopalatine fossa (PF) approaches employed for maxillary nerve (V2) involvement. Comparative studies in primary TN indicate comparable efficacy between these routes, with the PF approach offering shorter procedural duration (9, 10). However, evidence regarding recurrent TN management remains limited. Given potential anatomical alterations from prior interventions, this study prospectively evaluates the safety and efficacy of FO vs. PF approaches in 46 recurrent TN patients.

## Methods

### Study design and participants population

**Figure 1:**
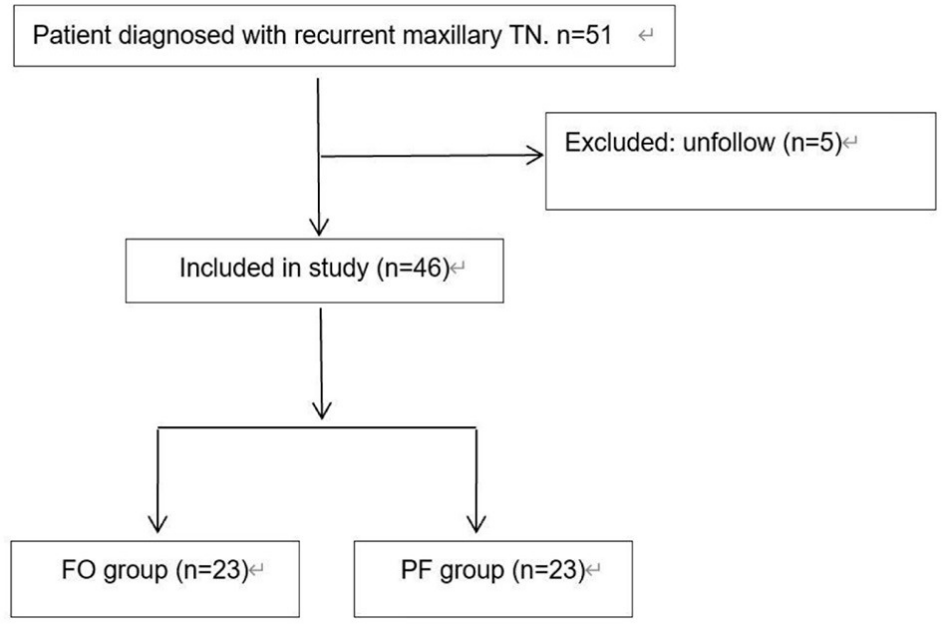
Flow chart of inclusion of trigeminal neuralgia patients.

This prospective non-randomized controlled trial enrolled 46 recurrent maxillary TN patients undergoing PRT at our institution between January and June 2021. The study protocol adhered to Declaration of Helsinki principles and received institutional ethics committee approval (2020-R562). Participants provided written informed consent and were allocated into FO (*n* = 23) or PF (*n* = 23) groups based on surgical approach. All procedures were performed under CT guidance by a single neurosurgeon. We provided patients with detailed explanations of both surgical approaches, allowing them to make an informed and voluntary choice between the FO and PF groups. Inclusion/exclusion criteria are detailed in Table 1, with patient flow illustrated in Figure 1.

**Table 1:** Inclusion criteria and exclusion criteria.

**Inclusion criteria**	**Exclusion criteria**
1. Definite diagnosis of recurrent maxillary TN.	1. Age below 30 years old or above 90 years old.
2. TN patients who had a history of TN related surgery, such as MVD, PRT and PBC.	2. Local infection of the puncture site.
3. Age between 30 and 90.	3. Coagulopathy or hemorrhagic disease.
4. VAS ≥ 4 points.	4. Severe heart, brain, lung and liver disease.
	5. Mental illness cannot cooperate with the operation.

TN, trigeminal neuralgia; VAS, visual analog score; MVD, microvascular decompression; PRT, percutaneous radiofrequency thermocoagulation; PBC, percutaneous balloon compression.

### Surgery

All interventions utilized local anesthesia with CT-guided stereotactic navigation. Patients received standard cardiovascular monitoring and intravenous access.

### FO approach

**Figure 2:**
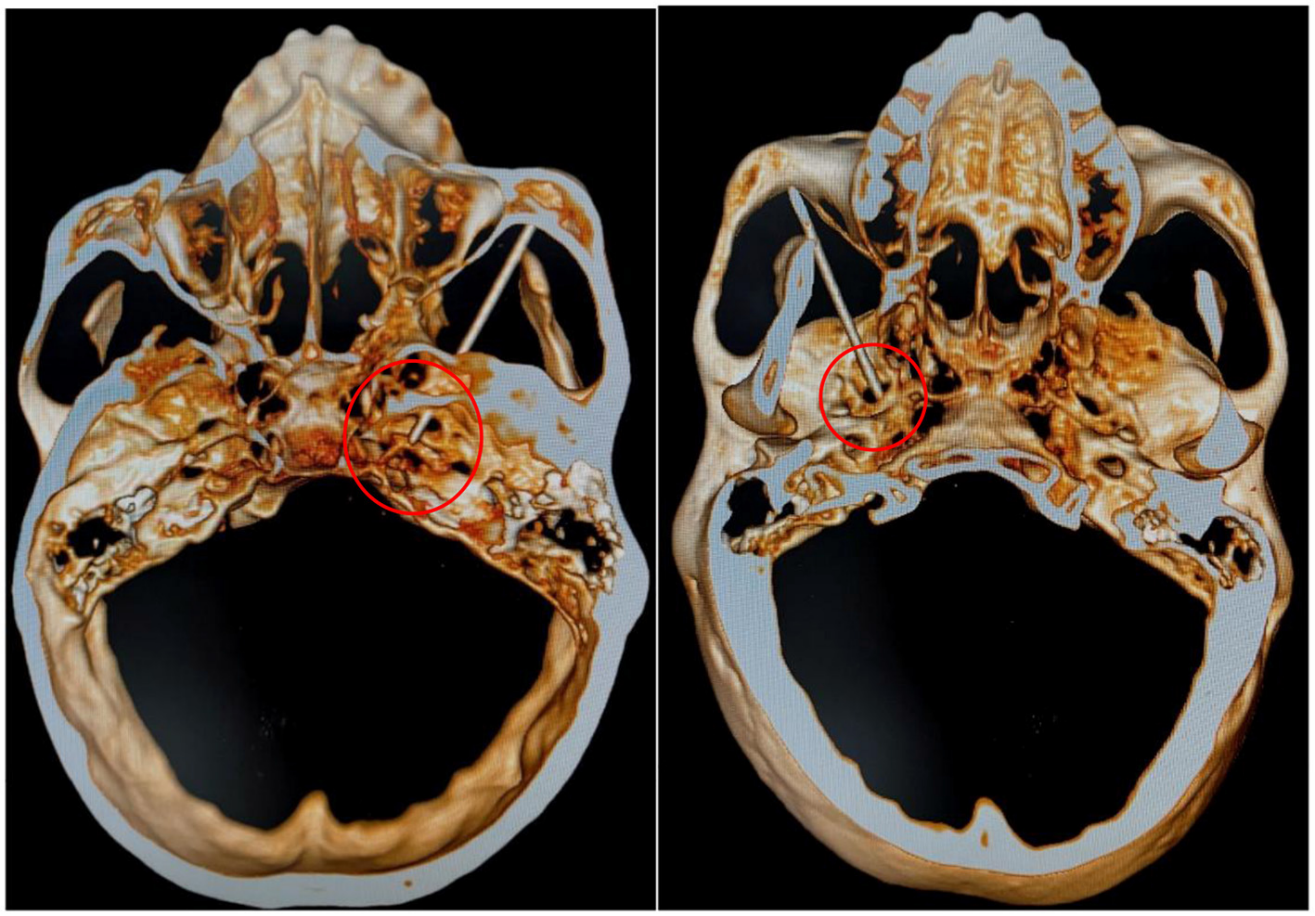
Under CT guidance, the precise position of the needle tip was confirmed. The puncture needle was advanced to the optimal depth within the foramen ovale, with meticulous efforts to avoid the mandibular nerve.

The position of operation was supine and connected to the ECG monitor. Blood pressure, heart and oxygen saturation were measured, and 0.03 mg/kg of midazolam was administered intravenously before surgery. A 10 cm, 22 G puncture needle was used to puncture the affected side, the radiofrequency machine was connected. Under the guidance of CT, the puncture needle entered the foramina ovale, and 50 Hz sensory stimulation and 2 Hz motor stimulation were given to induce sensation in the maxillary nerve region. An abnormal sensation or muscle contraction in the non-diseased area required adjustment of the tip position until the stimulated area covered the painful area, as shown in Figure 2. In addition, local anesthesia of 0.2 ml, 2% lidocaine injection was given. When the pain area became numb, the patient was given three 90sec radiofrequency thermocoagulation at 80°C.

### PF approach

**Figure 3:**
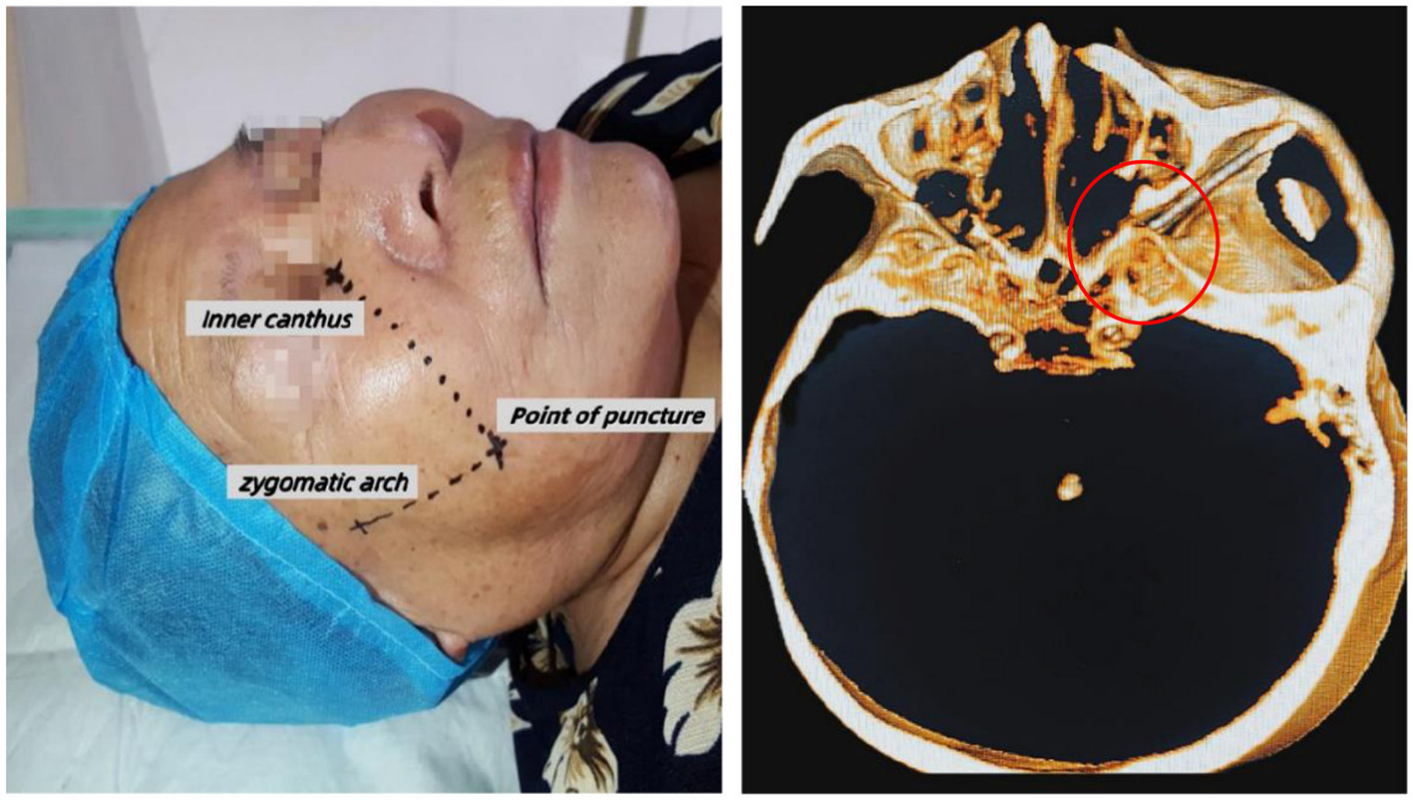
The puncture location was 0.5–1 cm below the junction of zygomatic bone and mandible. The puncture needle was successfully introduced into the pterygopalatine fossa.

**Figure 4:**
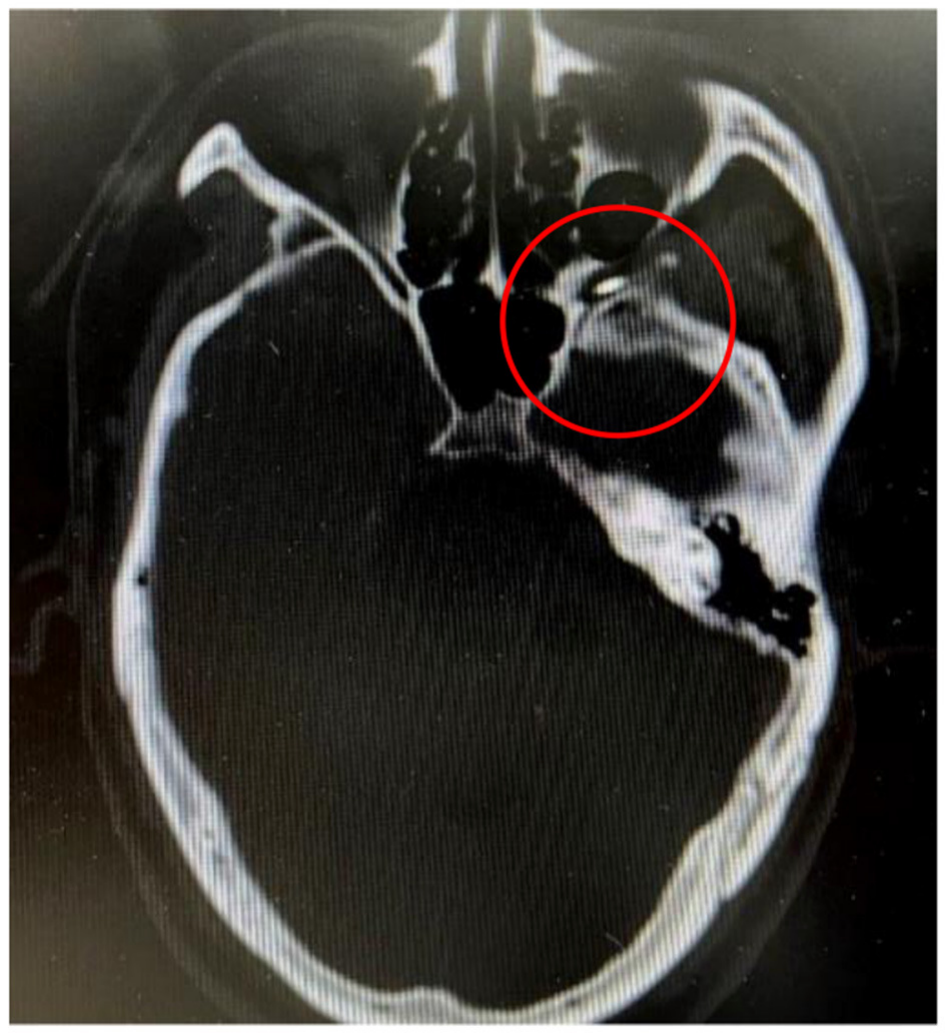
The needle tip has been accurately positioned at the foramen rotundum, however, it remains challenging to advance the needle tip into the foramen rotundum.

The patient remained awake, lying flat, and connected to a monitor to observe blood pressure, electrocardiogram, and blood oxygen saturation. Our puncture site was 0.5–1 cm below the junction of the zygomatic bone and mandible, as shown in Figure 3. Furthermore, midazolam (0.03 mg/kg) was injected intravenously for sedation before surgery. A 22 G local anesthesia needle, 10 cm in length, was used for a puncture, and 1% lidocaine (0.5 ml) was injected into the puncture path. In the CT-guided, the puncture needle arrives the foramen rotundum (FR). Maxillary nerve region sensation was induced by 50 Hz sensory stimulation and 2 Hz motor stimulation, as shown in Figure 4. Local anesthesia of 0.3 ml, 1% lidocaine injection was given. When the pain area became numb, the patient was given three 90-sec radiofrequency thermocoagulation at 85°C.

During the surgical procedure, we observed that in patients who had previously undergone percutaneous radiofrequency thermocoagulation (PRT) or percutaneous balloon compression (PBC), the impact of scarring on the operation was significant. This was evident in the tactile sensation during needle puncture and the administration of local anesthetic injection. Previous studies have demonstrated that within the Gasserian ganglion, the three major divisions of the trigeminal nerve (V1, V2, and V3) are in close proximity and partially interwoven. Consequently, when accessing the Gasserian ganglion via the foramen ovale (FO) approach, it is challenging to precisely locate the V2 division. In our experience with the FO approach, we attempted to avoid the mandibular nerve (V3). However, in the majority of patients, sensation in V3 was also elicited alongside V2 sensation.

### Therapeutic assessment and follow-ups

We utilized the Visual Analog Scale (VAS) and the Barrow Neurological Institute (BNI) Pain Intensity Scale for therapeutic assessment. Additionally, complications and pain recurrences were meticulously documented. The BNI Pain Intensity Scale was employed to evaluate pain severity in patients, with the results presented in Table 2. An outcome was deemed effective if the BNI Pain Score was categorized as I or II and the VAS score was < 4. Patients underwent follow-up evaluations at 6, 12, 18, and 24 months postoperatively, during which their pain and numbness were carefully recorded.

**Table 2:** Barrow Neurological Institute (BNI) pain intensity scale.

**Score and description**	
I	No pain, no medication
II	Occasional pain, not requiring medication
III	Some pain, adequately controlled with medication
IV	Some pain, not adequately controlled with medication
V	Severe pain, no pain relief

### Statistical analysis

Statistical analyses were performed using SPSS version 24.0 software. All variables were confirmed to follow a normal distribution. Quantitative variables are presented as mean ± standard deviation. Comparisons between groups were conducted using the paired samples *t*-test. Qualitative data are expressed as frequency and percentage (%). The threshold for statistical significance was set at *P* < 0.05.

## Results

### General demographics

In this study, the patients' ages ranged from 30 to 90 years, with a mean age of 70.91 ± 8.37 years. The difference in age between groups was not statistically significant (*P* = 0.435). The total disease duration varied from 6 to 480 months. The specific treatments received by each group of patients are detailed in Table 3. The time from initial diagnosis to surgery is shown in Table 4. All patients experienced pain localized exclusively to the maxillary division (V2) of the trigeminal nerve.

**Table 3:** Patient demographics and clinical data.

**Characteristics**	**Baseline (Mean ±SD)**
**Gender (** ***n*** **)**	
Male	14
Female	32
**Age year**	
Mean ± SD	70.91 ± 8.37
Range	31–89
**Pain duration, month**	
Range	6–480
**Pain side (** ***n*** **)**	
Right	31
Left	15
**Experience (** ***n*** **)**	
MVD	10
PBC	12
PRT	24
Preoperative VAS	Group FO: 7.43 ± 1.03
	Group PF: 7.26 ± 1.09

MVD, microvascular decompression; PBC, percutaneous Balloon Compression; PRT, percutaneous radiofrequency thermocoagulation; VAS, visual analog score.

**Table 4:** The time from initial diagnosis to surgery.

**Surgery**	**Group FO**	**Group PF**	***p*-value**
MVD	50.67 ± 17.72	48.75 ± 27.54	0.896
PBC	47.67 ± 21.44	54.50 ± 20.24	0.583
PRT	42.00 ± 19.53	41.15 ± 20.34	0.919

### Clinical outcomes

The efficacy rates of the two puncture pathways within 24 months were 69.5% (FO group) and 78.2% (PF group), respectively. There were no statistically significant differences in preoperative indicators between the two groups (age *P* = 0.435, gender *P* = 0.575, preoperative VAS *P* = 0.549, preoperative BNI *P* = 0.257). Blood pressure, heart rate, and oxygen saturation remained stable and showed no significant differences between the two groups before and after surgery. one patient (4.34%) in the FO group and three patients (13.0%) in the PF group continued to experience pain (VAS > 4); the same treatment was repeated 7 days later.

**Figure 5:**
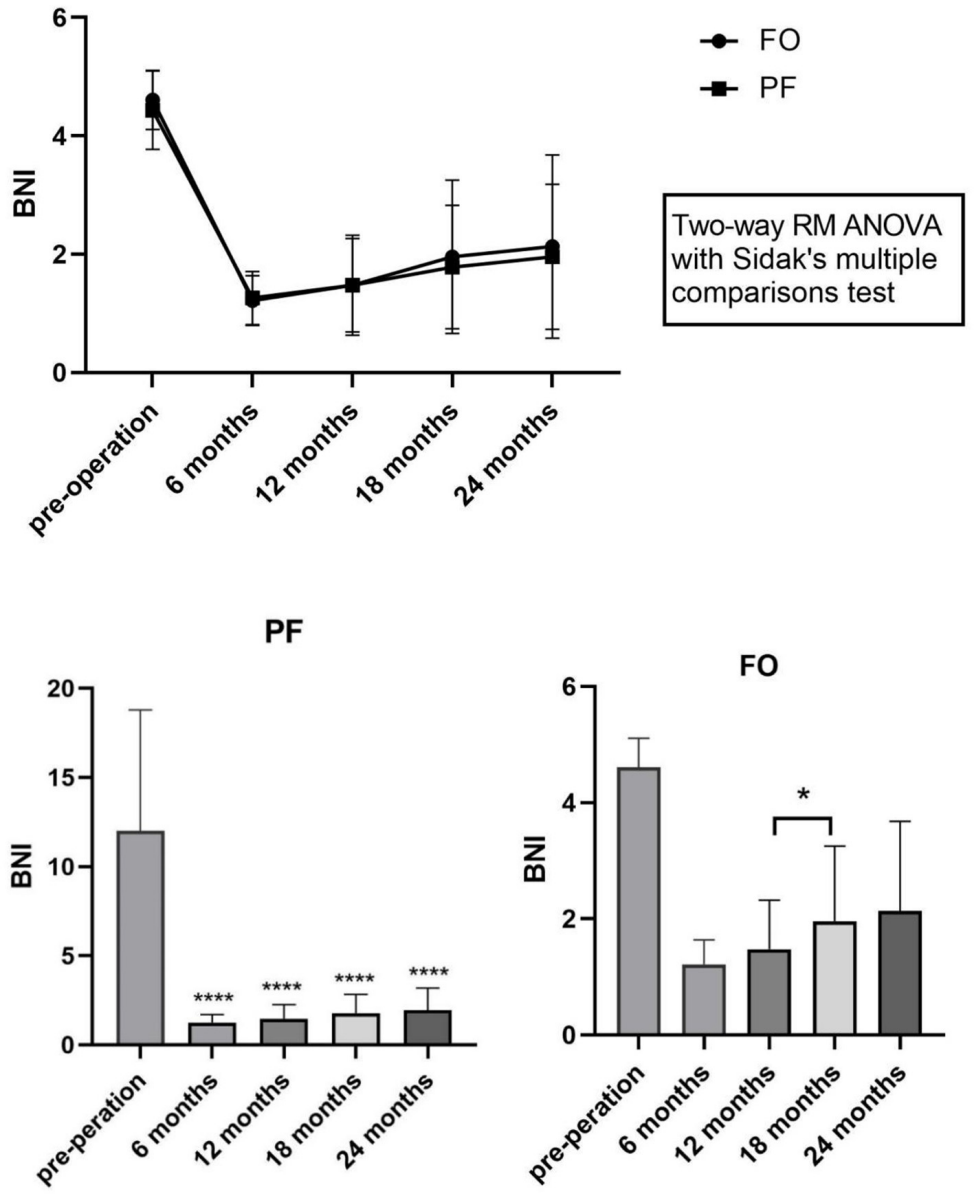
Post procedure BNI scores between two groups changes in BNI scores after percutaneous balloon compression compared with preoperative scores. ^*^P < 0.01, ^**^P < 0.05, ^***^P < 0.001, ^****^P < 0.0001. BNI, Barrow Neurological Institute; RM, repeated measurement; ANOVA, analysis of variance.

The effective rate of BNI (BNI I–II) in both groups reached 100% at 6 months postoperatively. At 12 months, 3 patients (13.0%) in the FO group and 2 (8.69%) in the PF group experienced pain recurrence. The number of recurrences in the two groups was 5 (21.7%) and three (13.0%) at 18 months, and seven (30.4%) and 5 (21.7%) at 24 months, respectively. Postoperative BNI scores in both the FO and PF groups were significantly reduced compared to preoperative scores, as illustrated in Figure 5.

The mean VAS scores for each time period in the FO group were 7.43 ± 1.03 preoperatively, 0.52 ± 0.66 at 6 months, 1.13 ± 1.35 at 12 months, 2.26 ± 2.66 at 18 months, and 2.78 ± 2.85 at 24 months. For the PF group, the mean VAS scores were 7.26 ± 1.09 preoperatively, 0.43 ± 0.66 at 6 months, 0.82 ± 1.33 at 12 months, 1.69 ± 2.30 at 18 months, and 2.43 ± 2.42 at 24 months. The comparison of VAS scores between the FO and PF groups during the follow-up period is presented in Table 5.

**Table 5:** Comparison of VAS score between the 2 groups.

**Follow-up time**	**Group FO**	**Group PF**	***p*-value**
6 months	0.52 ± 0.66	0.43 ± 0.66	0.604
12 months	1.13 ± 1.35	0.82 ± 1.33	0.110
18 months	2.26 ± 2.66	1.69 ± 2.30	0.331
24 months	2.78 ± 2.85	2.43 ± 2.42	0.618

### Side effects and complications

Postoperative hypoesthesia of the face and mucosa was observed in all patients. In the FO group, we attempted to avoid the mandibular nerve by placing the puncture as medial to the foramen ovale as possible. However, 17 patients (73.9%) still experienced numbness in the mandibular nerve area concurrent with the maxillary nerve area. Masseter muscle weakness occurred in six patients (26.0%), and headache was reported in four patients (17.3%), all from the FO group. The headache lasted for approximately 3 h and resolved spontaneously without the need for analgesic medication. In the PF group, two patients (8.69%) experienced diplopia, which raised concerns during the procedure. After confirming the needle tip position, it was determined that rapid injection of local anesthetic had caused it to enter the inferior orbital fissure and numb the extraocular muscles. Symptoms resolved within 1 h postoperatively in both patients. Facial swelling occurred in nine patients (39.1%) in the FO group and 11 patients (47.8%) in the PF group following treatment, likely due to vascular injury. No severe complications, such as blindness, intracranial hemorrhage, or intracranial infection, were observed in either group. Additionally, no deaths occurred during this study. A comparison of complication data is provided in Table 6.

**Table 6:** Comparison of complications between the two groups.

**Complications**	**Group FO**	**Group PF**
Facial hypoesthesia	100%	100%
Headache	17.3%	0
Masseter muscle weakness	26.0%	0
Diplopia	0	8.69%
Facial swelling	39.1%	47.8%

## Discussion

Trigeminal Neuralgia (TN) is a severe facial pain disorder, predominantly involving the V2 and V3 branches of the trigeminal nerve. It is typically unilateral, characterized by severe, episodic pain that significantly impairs patients' physical and mental health. Percutaneous radiofrequency thermocoagulation (PRT) is recognized as one of the most effective minimally invasive techniques for treating TN, with its safety, efficacy, success rate, and patient satisfaction widely acknowledged compared to balloon compression and microvascular decompression (MVD) (11).

The history of PRT dates back to 1931 when semilunar electrosurgical coagulation was first reported for TN treatment. Sweet and Wepsic (12) later modified this technique by introducing radiofrequency thermocoagulation targeting the semilunar ganglion, achieving favorable outcomes. Depending on the target—either the semilunar ganglion or peripheral nerves—different puncture pathways can be employed. Current research indicates that satisfactory results can be achieved regardless of the chosen pathway (13).

Recurrence remains a challenging issue in TN management. Although microvascular decompression (MVD) is effective, recurrent patients may be reluctant to undergo re-craniotomy, and MVD is often not the first choice for elderly patients, particularly those with comorbidities (14). We set the follow-up period at 2 years, which aligns with our previous study on PBC for recurrent TN. This enables us to better determine whether more suitable treatment options exist for recurrent TN. Our study demonstrates that PBC is an effective treatment for recurrent TN. However, for patients with isolated maxillary nerve distribution pain, some may decline PBC due to concerns about excessively extensive numbness. PRT allows for selective lesioning of the painful maxillary nerve branch and is associated with less postoperative discomfort compared to PBC. Regarding other treatments such as MVD or glycerol rhizotomy for recurrent TN, the relevant literature remains limited. Our 2-year follow-up study revealed that for recurrent TN, the efficacy rates of PRT via the two puncture pathways were 69.5% and 78.2%, respectively. No significant differences in postoperative efficacy were observed between the two groups. Several studies have investigated the recurrence rate of PRT in primary TN (15, 16), but data on the recurrence rate of PRT for recurrent TN are scarce. Our findings suggest that at 2 years, the recurrence rates in the foramen ovale (FO) group and pterygopalatine fossa (PF) group were 30.4% and 21.7%, respectively, which aligns with previous research.

Our prior studies demonstrated that balloon compression is a safe and effective treatment for recurrent TN, but its downside is the extensive numbness it may cause, making it less suitable for patients with isolated V2 pain (17). The advantage of PRT is its predictable effect and localized numbness. Although numbness is the most common complication of PRT, it is also a marker of successful treatment (18). In our study, all patients experienced facial numbness, but the extent varied depending on the puncture pathway. As many as 73.9% of patients in the FO group experienced numbness in both the V2 and V3 nerve regions, which is more extensive than that seen in the PF group. Studies have shown that foramen ovale puncture can sometimes interfere with the V1 or V3 nerves, leading to ocular complications or masseter muscle weakness (19). When considering only V2, the PF group may have an advantage over the FO group.

Temperature may not be the primary determinant of treatment efficacy. Research has shown that 68°C can effectively relieve pain while minimizing complications (20). Higher temperatures, above 65°C, are believed to selectively destroy Aδ and C pain fibers, which are more susceptible to heat damage than Aα and Aβ tactile fibers (21). Some scholars advocate a temperature of 75°C as optimal (22). In this study, we chose 75°C for the FO group, while the PF group was subjected to a higher temperature of 85°C. Higher temperatures have been shown to increase the degree of numbness, thereby reducing the recurrence rate (23). Our results indicate that the efficacy rate of the FO group was lower than that of the PF group.

Although FO puncture may theoretically pose a higher risk of serious complications such as intracranial hemorrhage, intracranial infection, and corneal ulcer (24), the use of three-dimensional (3-D) imaging reconstruction has been shown to produce more efficient and safer outcomes compared to two-dimensional imaging (25). No severe complications were observed in either group. In the PF group, the puncture needle could be more accurately placed near the foramen rotundum within the pterygopalatine fossa, effectively avoiding the sphenopalatine ganglion. It is worth noting that the pterygopalatine fossa is adjacent to the inferior orbital fissure, and overly deep puncture may cause damage to the muscles and nerves in this area. Two patients experienced diplopia following the injection of local anesthetic, likely due to rapid injection causing the drug to enter the inferior orbital fissure.

Operationally, FO puncture is relatively easier to perform. Due to the small foramen rotundum in the pterygopalatine fossa, the needle requires repeated adjustments to reach the target position, increasing the risk of surrounding tissue damage. In recurrent patients, scarring from previous procedures is a significant factor, affecting the feel of the puncture and the spread of anesthetic. For patients in the FO group who had previously undergone PRT or percutaneous balloon compression (PBC), the puncture resistance was often greater than in those undergoing initial treatment, complicating needle direction adjustments. The effect of scarring may be less pronounced in the PF group, as we did not encounter significant puncture difficulties.

This study was limited by its single-center design, small sample size, and relatively short follow-up period of 24 months. Further research is needed to evaluate the long-term efficacy of the two puncture pathways in treating recurrent TN, ideally involving larger sample sizes, longer follow-up durations, and multicenter collaborations.

## Conclusion

Both the FO and PF puncture pathways for PRT are safe and effective for treating recurrent maxillary TN. However, for patients with a history of prior PRT or PBC targeting the Gasserian ganglion, the PF approach may be preferable.

## Data Availability

The raw data supporting the conclusions of this article will be made available by the authors, without undue reservation.
